# miRNA sequencing reveals hypothalamic microRNAs associated with seasonal reproduction in Sunite sheep

**DOI:** 10.3389/fvets.2026.1774514

**Published:** 2026-04-21

**Authors:** Yajing Shao, Xiaoyun He, Zizhen Ren, Xin Li, Ran Di, Xiangyu Wang, Mingxing Chu, Yuliang Wen

**Affiliations:** 1 College of Animal Science and Veterinary Medicine, Henan Institute of Science and Technology, Xinxiang, China; 2 State Key Laboratory of Animal Biotech Breeding, Institute of Animal Science, Chinese Academy of Agricultural Sciences, Beijing, China

**Keywords:** hypothalamus, miRNA, photoperiod, seasonal estrus, sheep

## Abstract

The hypothalamus plays a vital role in reproductive processes. Previous studies have identified hypothalamic miRNAs involved in mammalian reproduction, but the miRNA profiles induced by photoperiod changes remain unclear. In this study, we investigated the effects of photoperiod on hypothalamic microRNA (miRNA) regulation by analyzing miRNA expression patterns in Sunite sheep under three different photoperiod conditions—short photoperiod (SP), short-to-long photoperiod transition (SLP), and long photoperiod (LP) —with three biological replicates per group, using miRNA-sequencing technology. A total of 57, 47, and 12 differentially expressed miRNAs (DEMs) were revealed between SP vs. LP, SP vs. SLP, and LP vs. SLP, respectively. We constructed a co-expression network of DEMs and their target genes, identified oar-miR-370-5p, oar-miR-10b, and oar-miR-3957-3p as the miRNAs with the highest numbers of predicted target mRNAs based on interaction analysis. Functional annotation analysis demonstrated that the target genes of DEMs were enriched in multiple KEGG pathways, among which the most significant pathways associated with reproduction included the GnRH signaling pathway, prolactin signaling pathway, VEGF signaling pathway, and Wnt signaling pathway. These findings elucidate the dynamic changes in hypothalamic miRNA expression under varying photoperiods and provide crucial insights into the molecular mechanisms of underlying seasonal estrus in sheep, laying a foundation for future functional validation studies.

## 1 Introduction

Reproductive efficiency constitutes a fundamental economic parameter in sheep production systems, wherein seasonal estrus imposes significant biological constraints. Seasonal estrus patterns in sheep are classified into long-day (LD) and short-day (SD) types based on photoperiod responsiveness (1). Decreasing daylight duration strongly stimulates these processes (2). This reproductive cycle is precisely regulated through photoperiod-induced neuroendocrine pathways mediated by the hypothalamic–pituitary-gonadal (HPG) axis, where seasonal variations in daylight duration modulate reproductive activity (3). Significant genetic variation exists in estrous characteristics among sheep breeds. Notably, Small-tailed Han sheep carrying the FecB mutation (a variant of the *BMPR1B* gene) demonstrate seasonal estrus with annual lambing rate exceeding 250%, while photoperiod-sensitive breeds like Sunit sheep maintain strict seasonal breeding under short-day conditions, achieving only a 112% lambing rate (4). Photoperiod signals are transmitted via the retina, suprachiasmatic nucleus (SCN), and pineal gland axis, which constitutes the upstream regulatory cascade of the hypothalamic–pituitary-gonadal (HPG) axis that directly controls reproductive activity. This transmission drives rhythmic melatonin secretion, which serves as the core molecular mediator linking external photoperiod signals and endogenous reproductive endocrine activity: melatonin can specifically bind to MT1 receptors in hypothalamic neurons, and its secretion duration is directly determined by the length of the dark period, thus encoding photoperiod information to regulate the activity of the HPG axis (5, 6). Under short photoperiods, prolonged melatonin secretion activates hypothalamic TSH signaling through MT1 receptors. This activation stimulates T4-to-T3 conversion via type 2 deiodinase (DIO2) in tanycytes. Ultimately, this cascade regulates pulsatile GnRH secretion from the hypothalamus, further promotes the secretion of luteinizing hormone (LH) and follicle-stimulating hormone (FSH) from the pituitary gland, and triggers gonadal development and estrus activity, completing the entire photoperiod-regulated reproductive axis response (7, 8). These well-characterized regulatory pathways provide crucial insights into the molecular mechanisms governing seasonal estrus and highlight potential targets for overcoming photoperiodic constraints on ovine reproductive efficiency in sheep.

MicroRNAs (miRNAs), key post-transcriptional regulators of gene expression, have emerged as crucial modulators of gene silencing and transcriptional control (9). In most mammals, miRNAs bind to the 3′ untranslated region (3′UTR) of target mRNAs via seed sequence complementarity, leading to translational repression (10). Growing evidence underscores the importance of miRNAs in animal reproduction, particularly in hypothalamus and pituitary regulation. For instance, the miR-9 and miR-200 families influence GnRH neuron migration, while let-7 modulates pubertal onset (11, 12). In sheep, miRNAs regulate reproductive performance by modulating gene expression within the HPG axis. Studies demonstrate that differentially expressed miRNAs target the thyrotropin-releasing hormone (TRH) and transthyretin (TTR) genes. By targeting these genes, the miRNAs influence GnRH secretion and reproductive processes (13, 14). Using high-throughput sequencing, Lei identified differentially expressed miRNAs and their target genes in the sheep hypothalamus. This analysis compared breeding and non-breeding seasons. The findings provide valuable insights into seasonal estrus regulation. Notably, hypothalamic miRNA expression profiles exhibit significant changes under different photoperiods. For example, 145 differentially expressed miRNAs were identified under long photoperiod conditions (15). These miRNAs potentially regulate reproductive function through pathways such as GnRH signaling, further emphasizing their role in reproductive physiology. Recent studies have expanded this understanding across multiple reproductive tissues. Liu et al. (13) profiled the sheep hypothalamus and pituitary under varying photoperiods, identifying differentially expressed miRNAs that contribute to photoperiodic regulation of the reproductive axis. Zhang et al. (16) integrated miRNA expression data from the uterus of seasonal estrus sheep, revealing that photoperiod changes affect uterine miRNA profiles and modulate pathways related to cell adhesion, proliferation, and apoptosis. Functional studies have further linked specific miRNAs to reproductive traits: miR-370-3p regulates reproductive characteristics by targeting *COL4A3* (17), while miR-25 influences expression of the seasonal estrus gene *CHGA* (1). However, the precise mechanisms by which hypothalamic miRNAs influence seasonal reproduction remain poorly understood.

While numerous studies have investigated miRNA dysregulation in human hypothalamic pathologies, their roles in normal physiological conditions, particularly in seasonal estrus regulation in sheep, have received limited attention (18–20). Sunite sheep is a typical photoperiod-sensitive short-day estrus breed native to Inner Mongolia, China. Compared with perennial estrus sheep breeds (such as Hu sheep and Small-tailed Han sheep) and weakly seasonal estrus breeds, Sunite sheep has an extremely strict seasonal reproductive pattern: under natural feeding conditions, its estrus activities are completely concentrated in the short-day season (September to December), and the anestrus period lasts for more than 8 months per year, with significant differences in reproductive hormone levels and estrus performance between breeding and non-breeding seasons (4). This highly typical seasonal estrus phenotype makes Sunite sheep an ideal model for dissecting the molecular regulatory mechanism of photoperiod-induced seasonal reproduction. To systematically explore miRNA function in seasonal reproduction, this study employed Sunite sheep as a model, performing hypothalamic transcriptome sequencing to analyze miRNA expression patterns under varying photoperiods. By integrating existing research, we constructed miRNA-mRNA co-expression networks and conducted functional enrichment analyses (Gene Ontology and KEGG pathways). These findings elucidate the regulatory significance of hypothalamic miRNAs in ovine seasonal estrus and establish a theoretical foundation for future research in this field.

## 2 Materials and methods

### 2.1 Experimental animals and sample collection

Nine healthy 3-year-old, non-pregnant Sunite ewes (body weight: 37 ± 0.78 kg) were selected from Wulate Middle Banner, Bayannur City, Inner Mongolia Autonomous Region, China, all of which had a history of at least three lambings and were subsequently raised at the Tianjin Institute of Animal Science, where they were provided with ad libitum feed and free access to water.

The total experimental period was 84 days. The experimental design comprised three groups (*n* = 3 per group): (i) SP42: short photoperiod (8 L:16D, lights on 10:30–18:30) for 42 days; (ii) LP42: long photoperiod (16 L:8D, lights on 6:30–22:30) for 42 days; and (iii) SLP42: transition from short to long photoperiod (8 L:16D for 42 days, then 16 L:8D for 42 days, sampled at day 84). Light intensity was maintained at ~350 lux during light phase and <5 lux during dark phase. The 42-day duration was selected based on previous studies demonstrating sufficient time for photoperiod-induced neuroendocrine changes in the ovine hypothalamus (21).

All ewes underwent bilateral ovariectomy (OVX) to eliminate endogenous ovarian steroid feedback, thereby removing the confounding influence of cyclic hormonal fluctuations present in intact animals (13, 21). Following OVX, each ewe received a subcutaneous estrogen implant (1.5 cm *β*-estradiol packed in 2 cm silicone tubing), which stabilized serum estradiol levels at 7.23 ± 2.50 pg./mL. This controlled hormone replacement allowed us to isolate the specific effects of photoperiod on hypothalamic miRNA expression. Ewes were then transferred to light-controlled sheds for photoperiod treatment as previously described. Finally, after completing the experimental protocols, all animals were humanely euthanized via pentobarbital injection (60–100 mg/kg) under anesthesia, and the intact hypothalamic tissues were promptly collected for further analysis to ensure data accuracy and reliability.

### 2.2 Total RNA extraction and quality detection

Total RNA was extracted from 200 mg hypothalamic tissues using TRIzol reagent (Thermo Fisher Scientific, v1.2) following the manufacturer’s standard protocol: tissues were first fully homogenized into powder in liquid nitrogen, then lysed in TRIzol reagent for 5 min at room temperature, followed by chloroform phase separation, isopropanol precipitation, and 75% ethanol washing to obtain purified total RNA (22, 23). RNA integrity was initially assessed by 1% agarose gel electrophoresis, where distinct 28S/18S rRNA bands with no obvious degradation were observed for all samples. RNA concentration and purity (A260/A280: 1.8–2.0) were measured via NanoDrop 2000 (v3.8). RNA quality was further rigorously assessed using an Agilent 2100 Bioanalyzer (v2.0), and only samples meeting the manufacturer’s recommended standard (RIN ≥ 7.5) were included in subsequent transcriptomic analyses.

### 2.3 Sequencing library construction and data analysis

For each experimental group, 3 independent biological replicates were used for small RNA library construction and high-throughput sequencing, with no technical replicates set. Total RNA (3 μg) was processed using the NEB Next^®^ Ultra™ Directional RNA Library Prep Kit for Illumina (v2.0) according to the manufacturer’s protocol. Small RNA libraries were constructed and sequenced on the Illumina HiSeq 4000 platform (150 bp paired-end) with HiSeq Control Software (HCS v3.3.8).

### 2.4 Identification and differential expression analysis of miRNAs

Raw sequencing data in FASTQ format were generated, and high-quality clean reads were obtained by removing adapter-contaminated reads, poly-N sequences, and low-quality reads (Phred score <30), followed by calculating Q30 scores and GC content using FastQC (v0.12.1). Subsequently, taxonomic annotation was performed, and miRNA expression was analyzed by aligning the reads to the reference genome Oar_v4.0 using Bowtie2 (v2.5.1). Novel miRNAs were predicted using miRDeep2 (v2.0.1.3) based on characteristic hairpin structures, minimum free energy, and read mapping patterns. For differential miRNA expression analysis, DESeq2 (v1.8.3) was employed with stringent thresholds of *p* ≤ 0.05 and |log₂(fold change)| ≥ 0 to identify significantly regulated miRNAs. Target Gene Prediction of miRNAs.

Based on our previous study (4), the differentially expressed gene (DEG) profiles of seasonal estrus sheep were obtained. The target genes of miRNAs identified in this study were predicted using three computational tools: miRanda (v3.3a), PITA (v6), and RNAhybrid (v2.1.2). To enhance reliability, only genes consistently predicted by at least two algorithms were retained as high-confidence miRNA targets.

### 2.5 Co-expression network analysis

The co-expression networks of differentially expressed (DE) miRNAs and their target DE mRNAs were constructed using Cytoscape software (v3.1.1). This integrative analysis aimed to identify hub miRNAs and elucidate their functional roles in seasonal estrus regulation (24).

### 2.6 Integrated functional annotation and enrichment analysis of differentially expressed miRNAs and mRNA targets

The differentially expressed genes (DEGs) and the predicted target genes of differentially expressed miRNAs (DEMs) were subjected to Gene Ontology (GO) and Kyoto Encyclopedia of Genes and Genomes (KEGG) pathway enrichment analyses using clusterProfiler (v4.6.2). The GO analysis covered three categories: biological process, molecular function, and cellular component. GO terms or KEGG pathways with a hypergeometric *p*-value < 0.05 were considered significantly enriched.

## 3 Results

### 3.1 Hypothalamic tissue sequencing data

Global miRNA expression profiles of the hypothalamus were characterized in Sunite ewes exposed to different photoperiods. Following stringent quality control procedures, all samples yielded clean reads with a minimum output of 0.566 Gb per sample, demonstrating high sequencing quality (Q30 scores ≥ 94.64%). The GC content across sequencing data showed consistent values between 48.70 and 49.79%, aligning with expected genomic GC distribution patterns. Subsequent data processing effectively removed adapter-contaminated reads, poly_N sequences (≥ 10%), and low-quality bases, resulting in ≥ 97.51% of clean reads from each library being accurately mapped to the ovine reference genome (Oar_v4.0) (Table 1).

**Table 1:** Summary of RNA-seq data.

Sample name	Clean reads (rate)	Clean bases (G)	Q30 (%)	GC content (%)	Total mapped (mapping rate)
SP42a	34,759,236 (98.28%)	0.765G	94.88%	48.88%	31,002,229 (92.39%)
SP42b	29,596,818 (98.03%)	0.651G	95.52%	48.97%	26,258,604 (92.53%)
SP42c	25,715,010 (97.51%)	0.566G	95.14%	49.55%	22,610,739 (92.04%)
LP42a	37,046,435 (98.33%)	0.815G	94.80%	49.64%	32,344,202 (91.06%)
LP42b	36,272,628 (98.51%)	0.798G	94.64%	49.54%	32,011,400 (91.70%)
LP42c	36,247,190 (98.16%)	0.797G	94.72%	49.79%	32,012,235 (91.12%)
SLP42a	32,375,939 (98.30%)	0.712G	94.77%	49.75%	28,483,376 (90.39%)
SLP42b	32,032,877 (98.48%)	0.705G	95.05%	49.63%	28,417,769 (91.50%)
SLP42c	32,778,560 (98.60%)	0.721G	94.86%	48.70%	28,876,669 (91.39%)

**Figure 1:**
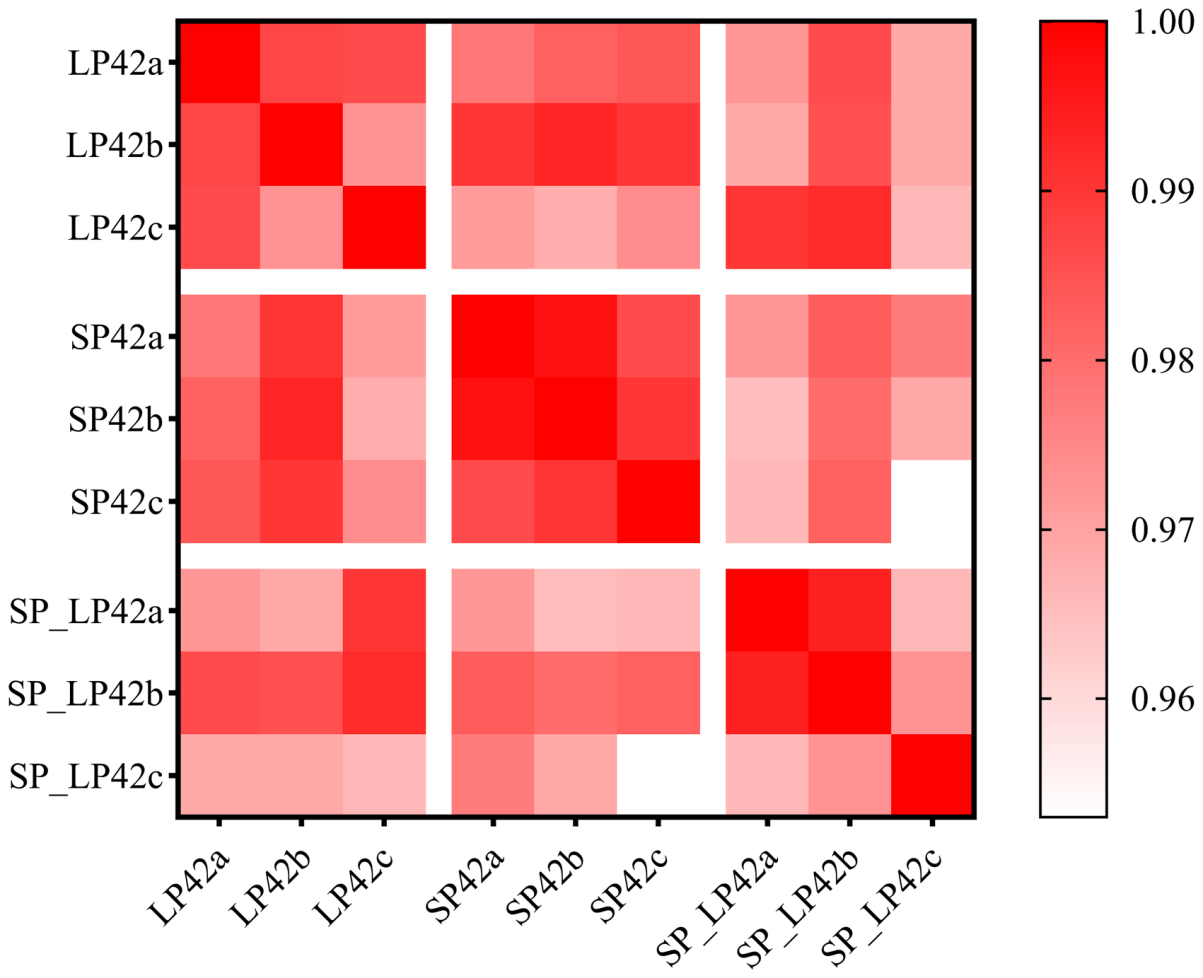
Correlation heatmap of sample pairs. This heatmap illustrates the Pearson correlation coefficients between different sample pairs. The color gradient ranges from light pink (low correlation) to dark red (high correlation), with a scale on the right indicating correlation values from 0.96 to 1.00. Diagonal elements, representing self-correlations, are colored in dark red. Sample labels: LP42, long photoperiod (16 L:8D) for 42 days; SP42, short photoperiod (8 L:16D) for 42 days; SP_LP42, short-to-long photoperiod transition (8 L:16D for 42 days followed by 16 L:8D for 42 days). Suffixes a, b, and c indicate three biological replicates per group.

To evaluate the consistency of triplicate transcriptome datasets for each sample, Pearson correlation coefficients were calculated. The analysis revealed that all nine sample groups exhibited Pearson correlation coefficients > 0.966, confirming high reproducibility among the three biological replicates within each group (Figure 1). Pearson correlation matrix for all biological replicates (Supplementary Table S1).

### 3.2 Identification of miRNAs in hypothalamic tissue

All filtered reads were systematically classified and annotated, encompassing known miRNAs, diverse non-coding RNAs (including rRNAs, tRNAs, snRNAs, snoRNAs, and other Rfam-classified ncRNAs), repetitive sequences, as well as small RNAs (sRNAs) that precisely align with mRNA exons/introns and novel miRNAs. Within each sample, less than 36% of reads matched known miRNA mature sequences, while no more than 40% matched other types of ncRNAs recorded in other RFAM databases (Table 2).

**Table 2:** Classification statistics of the total reads of sRNAs mapped to the reference genome.

Category	LP	LP (percent)	SP	SP (percent)	SLP42	SLP42 (percent)
Total	96,367,837	100%	79,871,572	100%	85,777,814	100%
known_miRNA	31,810,548	33.01%	28,599,304	35.81%	27,378,504	31.92%
rRNA	1,183,142	1.83%	273,213	0.53%	476,745	0.82%
tRNA	41	0.00%	30	0.00%	44	0.00%
snRNA	59,533	0.06%	19,182	0.02%	40,708	0.05%
snoRNA	337,271	0.32%	216,385	0.23%	360,217	0.36%
repeat	1,566,029	1.47%	1,228,312	1.31%	1,680,323	1.70%
novel_miRNA	248,572	0.24%	83,494	0.09%	223,802	0.23%
exon:+	9,571,611	9.16%	9,193,417	9.95%	9,242,608	9.51%
exon:−	41,888	0.04%	25,817	0.03%	42,391	0.05%
intron:+	18,513,813	19.38%	15,608,243	18.60%	18,212,755	20.57%
intron:−	764,401	0.99%	496,546	0.73%	705,322	1.00%
Other	32,270,988	40.62%	24,127,629	33.98%	27,414,395	37.26%

Furthermore, our analysis primarily focused on the identification of known and novel miRNAs. In total, we identified 349 miRNAs, including 197 known miRNAs and 152 novel miRNAs. The total clean reads annotated as known and novel miRNAs reached 87,788,356 and 555,868, respectively. These findings contribute to elucidating the role of miRNAs in gene expression regulation and biological processes. Notably, the discovery of novel miRNAs and subsequent analysis of their expression patterns may facilitate the identification of key regulatory nodes, offering potential biomarkers for the diagnosis and treatment of related diseases.

### 3.3 Differential expression analysis of miRNAs

**Figure 2:**
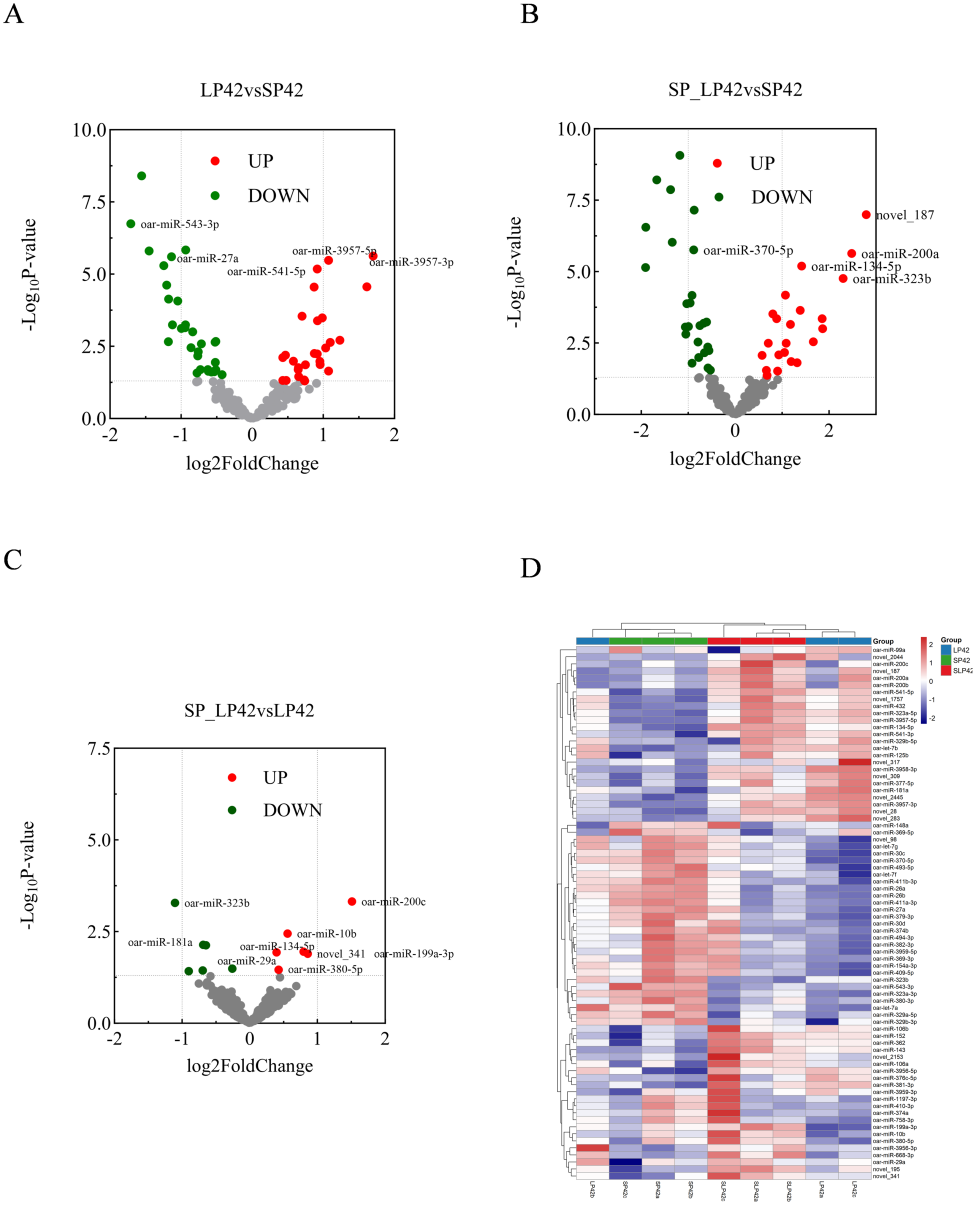
Presents three sets of differentially expressed metabolite (DEM) analyses. **(A–C)** Volcano plots showing significantly upregulated (red) and downregulated (blue) miRNAs in LP42 (long photoperiod, 42 days) vs. SP42 (short photoperiod, 42 days) **(A)**, SLP42 (short-to-long photoperiod transition, 84 days) vs. SP42 **(B)**, and SLP42 vs. LP42 **(C)**. The dashed line indicates the significance threshold (*p* < 0.05, |log₂FC| ≥ 0). Key differentially expressed miRNAs are labeled. **(D)** Heatmap showing the expression patterns of all significantly differentially expressed miRNAs (*p* < 0.05, |log₂FC| ≥ 0) across the three photoperiod groups (SP42, SLP42, LP42). Red indicates high expression, blue indicates low expression.

Compared to SP42, LP42 exhibited 57 DEMs (27 up-regulated, 30 down-regulated), while SLP42 versus SP42 showed 47 DEMs (22 up, 25 down). Between SLP42 and LP42, 12 DEMs (6 up, 6 down) were identified (Figures 2A–C). Hierarchical clustering of all DEMs revealed group-specific expression patterns (Figure 2D). The heatmap showed the expression patterns of all statistically significant differentially expressed miRNAs (*p* < 0.05) across the three photoperiod groups (Figure 2D). The heatmap of all DEMs is provided in Supplementary Figure S1. All differentially expressed miRNAs with log2FC and FDR (*p* < 0.05) (Supplementary Table S2).

### 3.4 miRNA-target gene network analysis

**Figure 3:**
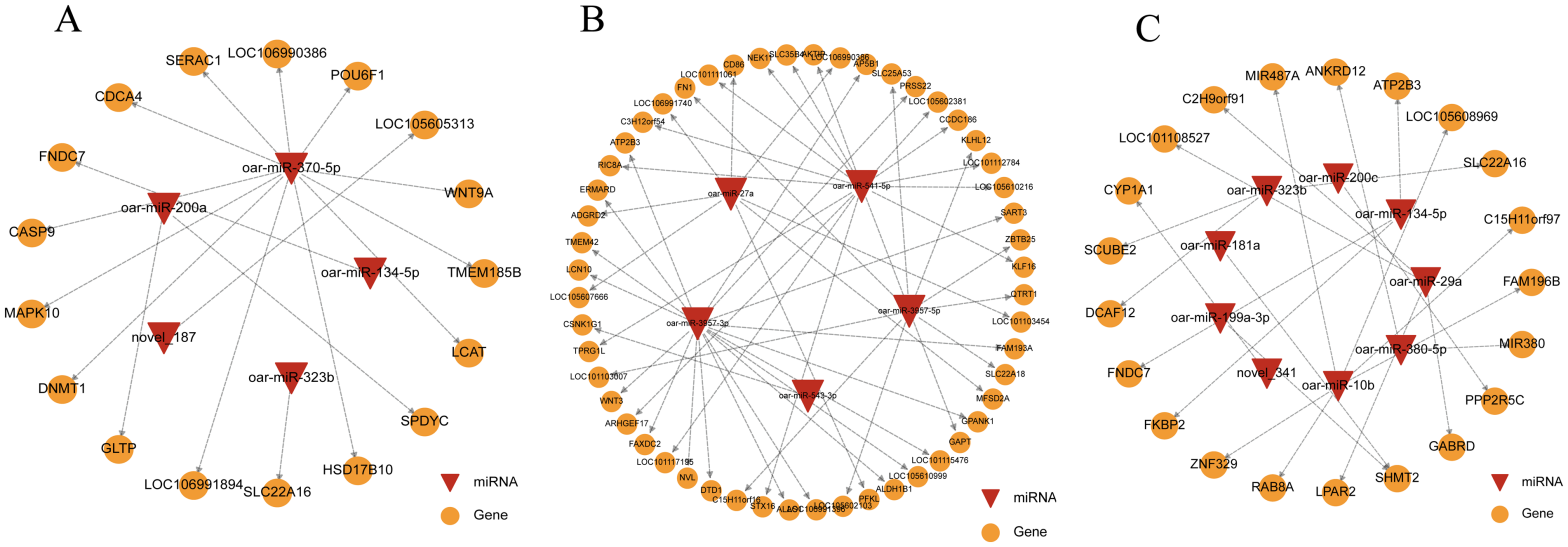
miRNA–mRNA interaction networks of differentially expressed miRNAs (DEMs) and their target genes. **(A–C)** Networks showing interactions between differentially expressed miRNAs and their target genes in SLP42 vs. SP42 **(A)**, LP42 vs. SP42 **(B)**, and SLP42 vs. LP42 **(C)**. Triangles represent significantly differentially expressed miRNAs, circles represent target genes, and edges indicate miRNA-target gene regulatory relationships. Only the top 10 most significant DEMs from each comparison group were included in the network analysis.

To elucidate the roles of differentially expressed miRNAs (DEMs) in the hypothalamus of Sunite sheep, the target genes of all DEMs in the three control groups were predicted. The top 10 most significant DEMs from each comparison group and their corresponding target genes were selected to construct miRNA-mRNA co-expression networks (Figures 3A–C, Supplementary Table S3). In the SLP42 versus SP42group, 5 DEMs targeted 17 DEGs (Figure 3A); in LP42 versus SP42, 5 DEMs regulated 52 DEGs (Figure 3B); and in SLP42 versus LP42, 9 DEMs modulated 22 DEGs (Figure 3C). Among these DEMs, oar-miR-370-5p, oar-miR-10b, and oar-miR-3957-3p exhibited the most pronounced differential expression across comparisons. Notably, oar-miR-370-5p has been implicated in hormone signal transduction, while oar-miR-10b is involved in neuroendocrine signaling pathways, suggesting their potential roles in photoperiodic regulation of reproduction. In the seasonal reproductive regulation of Sunite sheep, oar-miR-370-5p, oar-miR-3957-3p, and oar-miR-10b modulate key genes such as *CASP9*, *DNMT1*, *MAPK10*, *WNT3*, *ALDH1B1*, and *RAB8A*, thereby regulating apoptosis, DNA methylation, Wnt and MAPK signaling pathways, ultimately mediating photoperiod-driven seasonal changes in the estrous cycle and spermatogenesis.

### 3.5 Functional enrichment analysis of miRNA target genes

**Figure 4:**
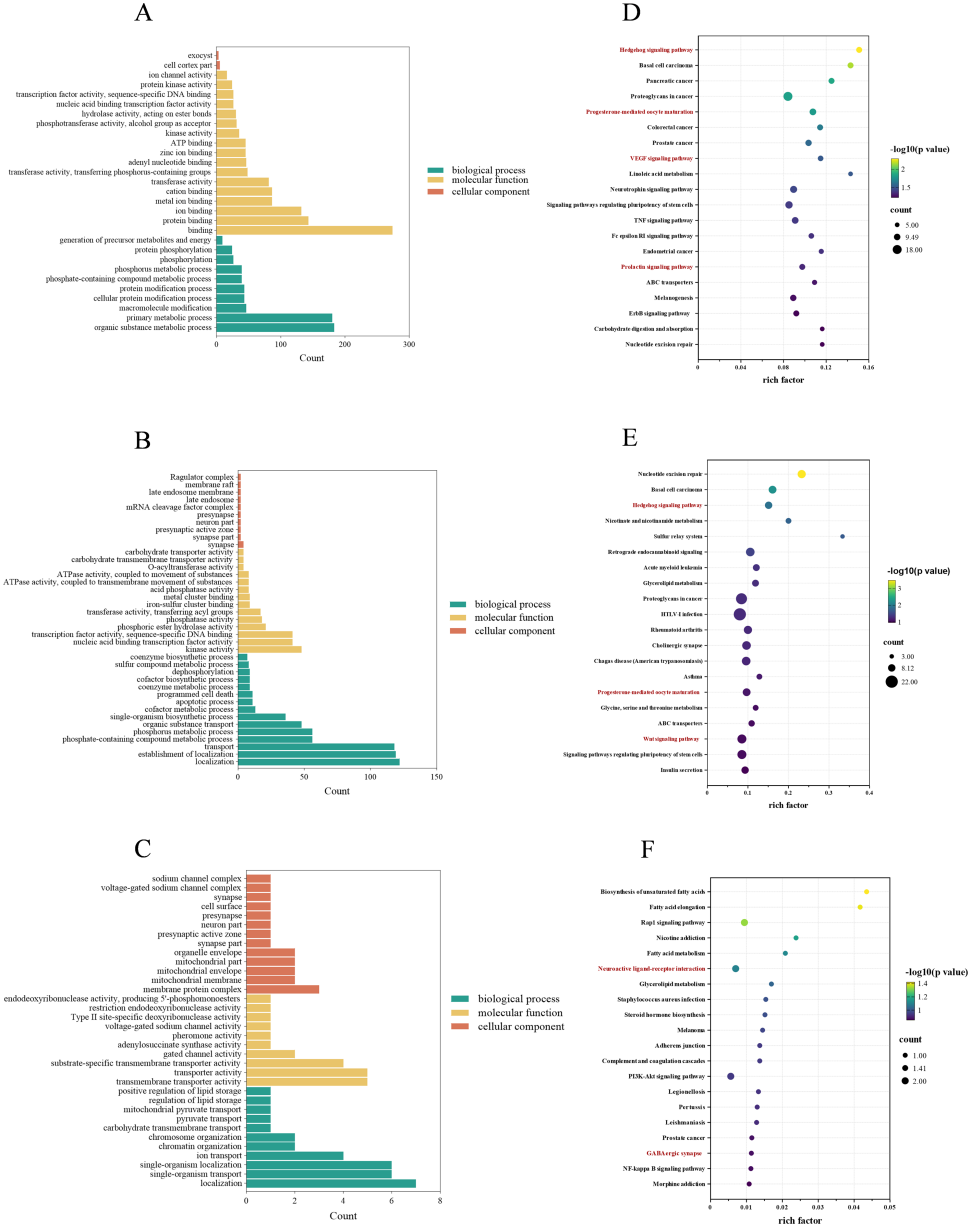
Enrichment analysis of GO terms and KEGG pathways for DEM-targeted genes across three comparison groups. **(A–C)** GO enrichment analysis for SLP42 vs. SP42 **(A)**, LP42 vs. SP42 **(B)**, and SLP42 vs. LP42 **(C)**. The *x*-axis represents the enriched GO terms, and the *y*-axis indicates the number of target genes. **(D–F)** KEGG pathway enrichment analysis for the same comparisons [**(D)**, SLP42 vs. SP42, **(E)**, LP42 vs. SP42, **(F)**, SLP42 vs. LP42]. In each bubble plot, the *x*-axis represents the rich factor (the ratio of enriched target genes to total genes in the pathway), the size of each bubble corresponds to the number of target genes (count), and the color gradient indicates the significance level (−log_10_ *p-*value). Pathways related to reproduction and neuroendocrine regulation are highlighted in red.

In the bioinformatic analysis of GO and KEGG pathways for the identified differentially expressed miRNAs (DEMs), GO enrichment revealed that the most significantly enriched terms in the biological process (BP) category across three comparison groups—SLP42 vs. SP42 (Figure 4A), LP42 vs. SP42 (Figure 4B), and SLP42 vs. LP42 (Figure 4C)—were organic nitrogen compound metabolic process, localization, and bioadhesion, respectively. For cellular components (CC), the predominant terms were extracellular matrix, synapse part, and mitochondrial part. In molecular function (MF), the top enriched terms included protein binding, ATP binding, and protein targeting to the ER. KEGG pathway analysis indicated that DEM target genes were significantly enriched in multiple functional pathways, among which the core pathways directly related to reproductive and neuroendocrine regulation included prolactin signaling pathway, GnRH signaling pathway, progesterone-mediated oocyte maturation and neuroactive ligand-receptor interaction, and other enriched pathways also contained VEGF signaling pathway, Hedgehog signaling pathway, etc. (Figures 4D–F, Supplementary Table S4). These enrichment patterns are likely closely associated with neural signal transduction, suggesting their critical roles in regulating reproductive behaviors and hormone secretion.

## 4 Discussion

The seasonal estrus is a critical adaptive mechanism that ensures optimal offspring survival and species continuity by synchronizing reproduction with favorable environmental conditions (25). The hypothalamus serves as the main control center for reproduction. It regulates the pulse release of gonadotropin-releasing hormone via neuroendocrine pathways, which then drives luteinizing hormone and follicle-stimulating hormone secretion (26, 27). Yet how the hypothalamus controls seasonal estrus at the molecular level remains poorly understood. Here we identify hypothalamic miRNAs linked to seasonal estrus in sheep and map their target gene networks. These results offer fresh insight into how day length regulates reproduction.

Our study identified 349 miRNAs across three control groups, with novel miRNAs representing 43.55% of the total. Comparative analysis revealed significant differential expression patterns, with 47, 57, and 12 differentially expressed miRNAs (DEMs) detected in the SLP42 vs. SP42, LP42 vs. SP42, and SLP42 vs. LP42 comparison groups, respectively. Network analysis of the top 10 DEM-targeted genes highlighted oar-miR-370-5p, oar-miR-10b, and oar-miR-3957-3p. These miRNAs exhibited the most robust targeting relationships across all comparison groups. This finding suggests their potential regulatory roles in seasonal estrus. These miRNAs may potentially modulate the HPG axis through three distinct mechanisms: neuroendocrine signal transduction, gonadal steroidogenesis, and oocyte maturation. The seasonal estrus cycle is governed by environmental cues such as photoperiod and temperature, which ultimately influence GnRH secretion through the HPG axis via melatonin and kisspeptin signaling pathways. Our findings build upon previous research demonstrating that miR-370-5p inhibits ovine melanocyte proliferation through *MAP3K8* targeting (28), while expanding the understanding of hypothalamic regulation in this process.

All three miRNAs were detected in hypothalamic tissue and exhibited photoperiod-dependent expression patterns. Oar-miR-370-5p levels varied between short and long photoperiods and across estrous stages. This miRNA targets *PRKCA* and other genes involved in hypothalamic neuroendocrine signal transduction. Through this targeting, it activates the GnRH signaling pathway to promote GnRH and LH secretion, thereby affecting follicular maturation and ovulation (29–32). oar-miR-10b functions within the hypothalamic–pituitary-ovarian axis to regulate GnRH secretion through modulation of neuroendocrine signal transduction pathways, including G-protein signaling. This regulation directly impacts FSH and LH levels, consequently controlling follicular development and ovulation timing. Additionally, oar-miR-10b expression directly responds to melatonin signaling stimulated by photoperiodic stimuli, and transmits upstream light signal to the hypothalamus neuroendocrine system to regulate sex hormone release via modulating the GnRH signaling pathway (33, 34). Similarly, oar-miR-3957-3p was differentially expressed miRNA in the hypothalamic transcriptome. Previous work has demonstrated its elevated expression in the pituitary during the luteal phase and its regulation of gonadotropin secretion via *PRKCA* targeting (31). Notably, both oar-miR-370-5p and oar-miR-3957-3p target *PRKCA*. This shared targeting suggests convergent regulation of hormone signal transduction at different levels of the HPO axis. The present findings suggest that oar-miR-3957-3p may act at multiple levels of the HPO axis (35). It appears to integrate photoperiodic signals in the hypothalamus and modulate hormonal output in the pituitary. This dual role potentially coordinates seasonal reproductive processes in sheep.

In this study, oar-miR-370-5p, oar-miR-10b, and oar-miR-3957-3p were identified as showing the most robust differential expression across control groups. Based on their predicted targeting networks, these miRNAs may be involved in regulating the estrous cycle and spermatogenesis through distinct target genes. Further analysis of these predicted targeting networks revealed the following potential regulatory mechanisms. Oar-miR-370-5p is predicted to target *CASP9*, *DNMT1*, and *MAPK10*, which are implicated in apoptosis, DNA methylation, and MAPK signaling, respectively. During follicular maturation, this miRNA may potentially suppress granulosa cell apoptosis by targeting *CASP9*, thereby potentially extending the maturation period and ensuring follicles reach ovulatory competence at the appropriate time; *CASP9* downregulation is hypothesized to reduce follicular cell apoptosis and extends follicular maturation (36), which is critical for coordinating follicular development with the seasonal breeding cycle. Concurrently, oar-miR-370-5p targets *DNMT1*, which may modulate DNA methylation status, influencing estrogen receptor gene expression and consequently regulating estrogen levels and estrus timing (37), while its effects on *MAPK10* may regulate GnRH release, thereby affecting LH/FSH secretion from the anterior pituitary and influencing both ovulation and spermatogenesis (38, 39). These convergent mechanisms suggest that oar-miR-370-5p may function as a central regulator of the seasonal estrus cycle. Oar-miR-10b is predicted to target *RAB8A*, which plays a critical role in vesicular transport of hormone receptors, suggesting that this miRNA may influence hormone signal transduction by regulating receptor cell surface localization or endocytic recycling; it is also predicted to target *ZNF329*, a zinc finger protein implicated in transcriptional regulation of gonad development-related genes, indicating its potential role in modulating reproductive organ development at the transcriptional level (40, 41). Thus, oar-miR-10b may regulate follicular maturation and spermatogenesis by controlling intracellular hormone signaling trafficking and receptor expression. Oar-miR-3957-3p potentially influences reproductive function by targeting WNT3, *ALDH1B1*, and *TMEM42*: WNT3 targeting regulates the Wnt/*β*-catenin pathway, which plays a central role in gonadal cell proliferation, differentiation, and development; *ALDH1B1* targeting suggests this miRNA may affect steroid metabolic pathways, thereby influencing testosterone synthesis and spermatogenesis (42); and *TMEM42* is associated with membrane signal transduction (43). Although the specific targeting mechanisms of oar-miR-3957-3p require further validation, its regulation of Wnt signaling and steroid metabolism likely contributes to seasonal reproductive control. Taken together, these three photoperiod-responsive miRNAs form a clear regulatory cascade: photoperiod-driven melatonin fluctuations alter their expression, which further modulates GnRH and related neuroendocrine pathway activity via targeting key genes, ultimately regulating ewe reproductive hormone secretion and estrus. These findings clarify how photoperiod signals are transduced into reproductive endocrine outputs at the miRNA level.

The three core miRNAs identified in this study synergistically regulate the key pathways of seasonal reproductive regulation: oar-miR-370-5p and oar-miR-10b jointly regulate GnRH signal transduction, oar-miR-10b acts as a downstream responder of melatonin signal, and all three miRNAs are involved in the hypothalamic neuroendocrine regulation of HPG axis activity. Functional enrichment analysis of differentially expressed miRNA target genes revealed significant involvement in multiple reproductive signaling pathways, including VEGF, prolactin, GnRH, progesterone-mediated oocyte maturation, neuroactive ligand-receptor interaction, Hedgehog, and Wnt pathways, consistent with previous findings by Dardente et al. (44–53). Building on Dardente’s identification of photoperiod-regulated thyroid hormone (TH) and Wnt signaling components (WNT9 and WNT3) in seasonal reproduction (44), the present study expands these observations to include additional regulatory mechanisms. Our results suggest that VEGF signaling, previously shown to mediate hypothalamic vascular dysfunction under metabolic stress (45, 46), may similarly influence seasonal estrous cycles through neurovascular remodeling. The identified neuroactive ligand-receptor interactions align with established mechanisms regulating reproductive behavior and energy balance in seasonal breeders (47, 48), while Hedgehog pathway components (WNT2 and WNT9A) showed dynamic expression patterns consistent with their roles in endometrial remodeling and folliculogenesis (49). These findings parallel research in Jining Grey goat where hypothalamic miRNAs during sexual maturation predominantly targeted GnRH signaling pathway components (54), and broad GnRH receptor distribution across brain regions (hippocampus, amygdala) underscores its central role in reproductive neuroendocrinology (55, 56). Consistent with our findings, a previous study on Small Tail Han sheep (a seasonal estrus sheep breed) also found that oar-miR-370 family (annotated as oar-miR-370-3p in the original study, the homologous mature isoform of oar-miR-370-5p identified in our study) and oar-miR-10b were significantly differentially expressed in hypothalamus under long and short photoperiod treatments (51, 52), which further confirmed the conservation of these two miRNAs in regulating photoperiodic reproductive response among different sheep breeds. A notable finding is oar-miR-10b’s potential targeting of *CDC25B*—a critical regulator of progesterone-mediated oocyte maturation—revaealing a novel mechanistic link between miRNA regulation and follicular development. *CDC25B*’s established role in *CDK1* activation and G2/M transition during oocyte maturation (53, 57), coupled with its importance in progesterone signaling (52), suggests that miR-10b-mediated *CDC25B* suppression could disrupt these essential reproductive processes. This mechanism gains clinical relevance in miRNA dysregulation in progesterone-resistant conditions like endometriosis, where aberrant miR-29c-3p and miR-126-3p expression contributes to disease pathogenesis (58, 59). Collectively, these findings position oar-miR-10b as both a potential modulator of seasonal reproduction and a candidate therapeutic target for reproductive disorders, highlighting the complex interplay between hypothalamic miRNAs and multiple synergistic pathways in reproductive regulation. Several limitations should be acknowledged, including breed-specific effects and lack of functional validation, warranting cautious interpretation. Future investigations should incorporate multi-omics integration and mechanistic studies across diverse breeds to fully elucidate these regulatory pathways and their translational potential for both animal reproduction and human reproductive disorders.

## 5 Conclusion

In this study, we systematically characterized hypothalamic miRNA expression profiles in sheep under different photoperiodic conditions. Through differential expression analysis and comparative genomic approaches, we identified a core set of photoperiod-responsive miRNAs, including oar-miR-370-5p, oar-miR-10b, and oar-miR-3957-3p. These miRNAs are predicted to target genes involved in key neuroendocrine and reproductive pathways, such as GnRH signaling, Wnt signaling, and steroid metabolism, suggesting their potential roles in regulating seasonal estrus. These findings provide a foundation for understanding of the molecular mechanisms underlying photoperiod-dependent reproductive control in sheep and identify candidate targets for future functional validation studies, including miRNA overexpression or knockdown experiments and validation of miRNA-target gene interactions.

## Data Availability

The BioProject is now publicly accessible at: https://www.ncbi.nlm.nih.gov/bioproject/PRJNA1427922.

## Supplementary material

Supplementary Table 1 Summary of RNA-seq data.

Supplementary Table 2 Classification statistics of the total reads of sRNAs mapped to the reference genome.

Supplementary Table 3 Pearson correlation coefficients among the three biological replicates of the transcriptome materials.

Supplementary Table 4 Statistical summary of differentially expressed miRNAs (DEMs) across three pairwise comparative analyses.

Supplementary Table 5 Summary of miRNA-mRNA interaction pairs between differentially expressed miRNAs (DEMs) and their target genes.

Supplementary Table 6 Enrichment analysis results of GO and KEGG for DEM target genes in SLP42 vs. SP42, LP42 vs. SP42 and SLP42 vs. LP42 comparisons.

Supplementary Table 7 List of differentially expressed miRNAs with fold change, adjusted p-value (FDR) and corresponding predicted target genes.
